# Assessing the impact of patient and public involvement on recruitment and retention in clinical trials: a systematic review

**DOI:** 10.1186/1745-6215-16-S2-O91

**Published:** 2015-11-16

**Authors:** Joanna Crocker, Adwoa Hughes-Morley, Sophie Petit-Zeman, Sian Rees

**Affiliations:** 1 Health Experiences Institute, University of Oxford, Oxford, UK; 2 NIHR Oxford Biomedical Research Centre, Oxford, UK; 3 MRC North West Hub for Trials Methodology Research, Manchester Academic Health Science Centre, University of Manchester, Manchester, UK; 4 NIHR Oxford Biomedical Research Centre and Unit, Oxford, UK

## Background and aims

Patient and public involvement (PPI) is research being carried out ‘with’ or ‘by’ patients and/or members of the public rather than ‘to’, ‘about’ or ‘for’ them. Some evidence suggests that PPI in clinical trials may improve recruitment and retention. We undertook a systematic review to determine whether or not this is confirmed by quantitative evidence. We also explored whether the impact on recruitment and retention varies according to context and PPI method.

## Methods

The following data sources were searched: Medline, Science Citation Index, Social Science Citation Index, Embase, PsychINFO, CINAHL, the Cochrane Library, the Health Expectations journal, the INVOLVE Evidence Library, and international trial registers. Forwards and backwards citation searches were performed on all eligible papers. Eligible studies were those which quantitatively evaluated the impact of PPI on recruitment and/or retention in clinical trial(s) in any population. Data concerning the clinical trial(s), PPI methods, evaluation methods and reported impacts on recruitment and retention were extracted from eligible papers, and studies were critically appraised using Cochrane risk of bias tools for randomised and non-randomised studies.

## Results

A total of 4924 records have been screened, 99 full-text articles have been assessed for eligibility, and 32 eligible studies have been identified. These studies cover a diverse range of PPI methods and include both experimental and observational evaluations. Data synthesis is in progress and the preliminary findings will be presented, along with their implications for clinical trial design and conduct.

